# Heterogeneity of Equilibrium Molten Globule State of Cytochrome *c* Induced by Weak Salt Denaturants under Physiological Condition

**DOI:** 10.1371/journal.pone.0120465

**Published:** 2015-04-07

**Authors:** Hamidur Rahaman, Md. Khurshid Alam Khan, Md. Imtaiyaz Hassan, Asimul Islam, Ali Akbar Moosavi-Movahedi, Faizan Ahmad

**Affiliations:** 1 Centre for Interdisciplinary Research in Basic Sciences, Jamia Millia Islamia, Jamia Nagar, New Delhi, 110025, India; 2 Institute of Biochemistry and Biophysics, University of Tehran, Tehran, Iran; University of Pittsburgh School of Medicine, UNITED STATES

## Abstract

While many proteins are recognized to undergo folding via intermediate(s), the heterogeneity of equilibrium folding intermediate(s) along the folding pathway is less understood. In our present study, FTIR spectroscopy, far- and near-UV circular dichroism (CD), ANS and tryptophan fluorescence, near IR absorbance spectroscopy and dynamic light scattering (DLS) were used to study the structural and thermodynamic characteristics of the native (N), denatured (D) and intermediate state (X) of goat cytochorme *c* (cyt-*c*) induced by weak salt denaturants (LiBr, LiCl and LiClO_4_) at pH 6.0 and 25°C. The LiBr-induced denaturation of cyt-*c* measured by Soret absorption (Δ*ε*_400_) and CD ([*θ*]_409_), is a three-step process, N ↔ X ↔ D. It is observed that the X state obtained along the denaturation pathway of cyt-*c* possesses common structural and thermodynamic characteristics of the molten globule (MG) state. The MG state of cyt-*c* induced by LiBr is compared for its structural and thermodynamic parameters with those found in other solvent conditions such as LiCl, LiClO_4_ and acidic pH. Our observations suggest: (1) that the LiBr-induced MG state of cyt-*c* retains the native Met80-Fe(III) axial bond and Trp59-propionate interactions; (2) that LiBr-induced MG state of cyt-*c* is more compact retaining the hydrophobic interactions in comparison to the MG states induced by LiCl, LiClO_4_ and 0.5 M NaCl at pH 2.0; and (3) that there exists heterogeneity of equilibrium intermediates along the unfolding pathway of cyt-*c* as highly ordered (X1), classical (X2) and disordered (X3), i.e., D ↔ X3 ↔ X2 ↔ X1 ↔ N.

## Introduction

The folding from the readily synthesized unfolded protein at ribosome to the native active state is remarkably fast despite the astronomical number of possible conformations available to polypeptides. All the proposed mechanisms for protein folding, i.e., the framework model, the diffusion-collision model, the nucleation growth mechanism or the hydrophobic collapse model show that when a polypeptide folds into its native state, there is progressive stabilization of partially structured folding intermediates in a hierarchical manner to prevent the polypeptide chain to go through all possible conformations [1–5]. The characterization of the intermediates on folding pathways is very important as they can serve as initiation points of aggregation apparent in a number of diseases. Many globular proteins have been shown to adopt intermediate states that are molten-globule-like as observed from the classical perspective of protein folding [6–9].

The MG state generally characterized by the presence of native-like secondary structure and compactness with highly labile side chain, corresponds to late folding intermediate [10,11]. The equilibrium MG state was reported for the first time in cytochrome *c* (cyt-*c*) under special condition, such as low pH and high concentrations of anions [12–16]. MG states of a protein generated at low acidic and high alkaline pH have a wide range of structural and thermodynamic flexibility [16–19]. However, nature and range of fluctuations of the MG states of a protein under physiological condition and the mechanism that stabilizes them are not well studied. Most importantly, the MG state is important for protein function in living cells [20,21] especially in protein-DNA and protein-protein interactions [22–26]. Therefore, it will be important to evaluate the structural details of the MG states of proteins under physiological condition and understand the interactions that stabilize them.

Cyt-*c* is a model protein for the characterization of intermediate state of protein folding [27–29]. In the previous study we have reported that the weak salt denaturants such as LiCl, CaCl_2_ and LiClO_4_ induce MG state of horse and bovine cyts-*c* under physiological conditions [7,30]. The MG state induced by LiCl, CaCl_2_ and LiClO_4_ has the same thermodynamic stability though they differ in their structural properties [30]. The present study reports the structural and thermodynamic characteristics of the LiBr-induced MG state of goat cytochrome *c* (cyt-*c*) and compares them with those of MG states induced by LiCl, LiClO_4_ and low pH (2.0) in the presence of 0.5 M NaCl. Furthermore, this study reveals that LiBr induces a unique MG state on its folding ↔ unfolding pathway which is structurally different from MG states observed under different solvent conditions at pH 6.0 and 25°C.

## Materials and Methods

Fresh got heart was collected from the Slaughter House, Ghazipur Mandi, New Delhi. The cyt-*c* was isolated from goat heart according to the method described previously [31,32]. The protein was purified by gel filtration (90×2.3 cm) on Sephadex G-50 column chromatography. The purity was checked using sodium dodecyl sulfate polyacrylamide gel electrophoresis (SDS-PAGE) on which a single band was observed indicating the purity of the protein. The laboratory reagents such as LiCl, LiClO_4_, LiBr, ANS and sodium salt of cacodylic acid were purchased from Sigma Chemical Co. (U.S.A.). Sodium chloride and potassium chloride were purchased from Merck (India). These and other chemicals were analytical-grade reagents and were used without further purification.

### Preparation of Solutions

The cyt-*c* was oxidized first by adding 0.1% potassium ferricyanide using a procedure as described earlier [33]. The concentration of the oxidized cyt-*c* was determined experimentally using a value of 106 x 10^3^ M^-1^ cm^-1^ for the molar absorption coefficient (*ε*) at 409 nm [32]. All solutions were prepared in 30 mM cacodylate buffer containing 100 mM KCl (except 50 mM NaCl in case of LiClO_4_) at pH 6.0 for optical measurements and incubated overnight at room temperature.

### Absorbance and CD Measurements

Isothermal denaturation of cyt-*c* by LiBr, LiCl and LiClO_4_ at 25.0 ± 0.1°C and pH 6.0 was measured in Shimadzu 2100 UV/Vis spectrophotometer whose temperature was maintained by circulating water from an external refrigerated water bath. The thermal denaturation of the protein in the presence and absence of different concentrations of LiBr was carried out in Jasco J-715 UV/Vis spectrophotometer equipped with a Peltier-type temperature controller (PTC-348WI), with a heating rate of 1°C/min providing adequate time for equilibration. The change in the absorbance at 400 nm of the protein was measured in the temperature range 20–85°C. A total of about 500 data points were collected for each transition curve. Protein concentration used for the absorption measurements in the Soret and IR regions was in the range 7–10 *μ*M and 80 *μ*M, respectively. The fused quartz cell having 1.0 cm path length was used for the absorption measurements.

The CD instrument was routinely calibrated with D-10-camphorsulphonic acid. The results of all the CD measurements are expressed as mean residue ellipticity ([*θ*]_*λ*_) in deg cm^2^ dmol^-1^ at a given wavelength λ (nm) using the relation,

$${\left[\theta \right]}_{\lambda }=\theta_{\lambda }M_{0}/10cl$$ (1)

where *θ*_*λ*_ is the observed ellipticity in millidegrees at wavelength *λ*, *M*_0_ is the mean residue weight of the protein, *c* is the protein concentration (mg/cm^3^), and *l* is the path length (cm). It should be noted that each observed *θ*_λ_ of the protein was corrected for the contribution of the solvent. For CD measurements in the far-UV and Soret regions, 0.1- and 1-cm path length fused quartz cells were used, respectively, and protein concentration was in the range18–20 *μ*M. Reversibility of the isothermal denaturation by weak salts was checked using the procedure described earlier [34]. At a given denaturant concentration, the denaturation and renaturation experiments were measured and compared to check reversibility. Reversibility of the thermal denaturation was checked by matching the optical properties before and after denaturation.

### Infrared Measurements

Infrared spectra (IR) were measured with a Perkin- Elmer Model RX-I Fourier transforms infrared spectrophotometer at 25°C. Protein samples (10–12 mg/mL) were loaded in a heatable IR cell with CaF_2_ windows having 6 μm path length. For each spectrum, a 100-scan interferogram was collected in a double beam mode with a 2 cm^-1^ resolution from 1200 to 2000 cm^-1^. Reference spectra were recorded under identical conditions with only the media in which the protein was dissolved in the cell. The protein spectra were obtained according to water subtraction procedure [35–37].

### Fluorescence Measurements

Fluorescence measurements were performed in Jasco FP-6200/STR-312 spectroflouremeter in a quartz cell (Hellma) with a path length of 5 mm at 25°C, using 5 nm excitation and emission slits. Protein concentration for all the experiments was in the range 7–10 *μ*M. ANS (250 *μ*M) fluorescence spectra were collected from 400 to 600 nm with excitation at 360 nm. However, for tryptophan (Trp) fluorescence measurements the emission spectra were recorded in the wavelength region 300–400 nm after excitation at 282 nm.

### Dynamic light scattering (DLS) measurements

Dynamic light scattering measurements were carried out in a RiNA Laser Spectroscatter-201 to obtain hydrodynamic radii of different states of cyt-*c* at 25 ± 0.1°C. The protein samples were filtered through a 0.22 μm pore size filter paper (Millipore). Concentration of the protein used in DLS measurements was 2.5 mg/ml. Measurements were made at a fixed angle of 90° using an incident laser beam of 689 nm. Ten measurements were made with an acquisition time of 30 sec for each sample at a sensitivity of 10%. The data were analyzed using PMgr v3.01 p17 software provided by the manufacturer to get hydrodynamic radius, and polydispersity (the standard deviation of the size of the particle). These measurements are used to determine the compactness factor (CF) using the relation [38],

$$CF=(R_{D}-R_{MG})/(R_{D}-R_{N})$$ (2)

where *R*_D_, *R*_MG_ and *R*_N_ are hydrodynamic radii in nm of the denatured, intermediate and native states of the protein, respectively. The theoretical hydrodynamic volume of a single protein molecule (= 4/3π*R*_N_^3^) of cyt-*c* in the native state was obtained using the relation [33],

$$V=1.212\left(\frac{nm^{3}}{Da}\right)*10^{-3}*M\left(Da\right)$$ (3)

where *M* is the molecular mass of the protein in Dalton.

## Results

### Weak salt denaturants-induced denaturations of cyt-*c*

To determine denaturation curves of different optical properties, we measured visible absorption and CD spectra and far-UV CD spectra in the presence of different concentrations of LiBr, LiCl and LiClO_4_ at pH 6.0 and 25°C. These spectra are given in S1 Fig., S2 Fig., and S3 Fig. Denaturation induced by lithium salts was monitored by [*θ*]_222_ (probe for measuring change in backbone conformation), [*θ*]_416_ (probe for measuring change in heme-Met and Phe interactions), [*θ*]_409_ (probe for measuring change in heme-globin interaction), Δ*ε*_~400_ and Δ*ε*_409_ (probes for measuring change in heme-globin interaction).

**Fig 1:**
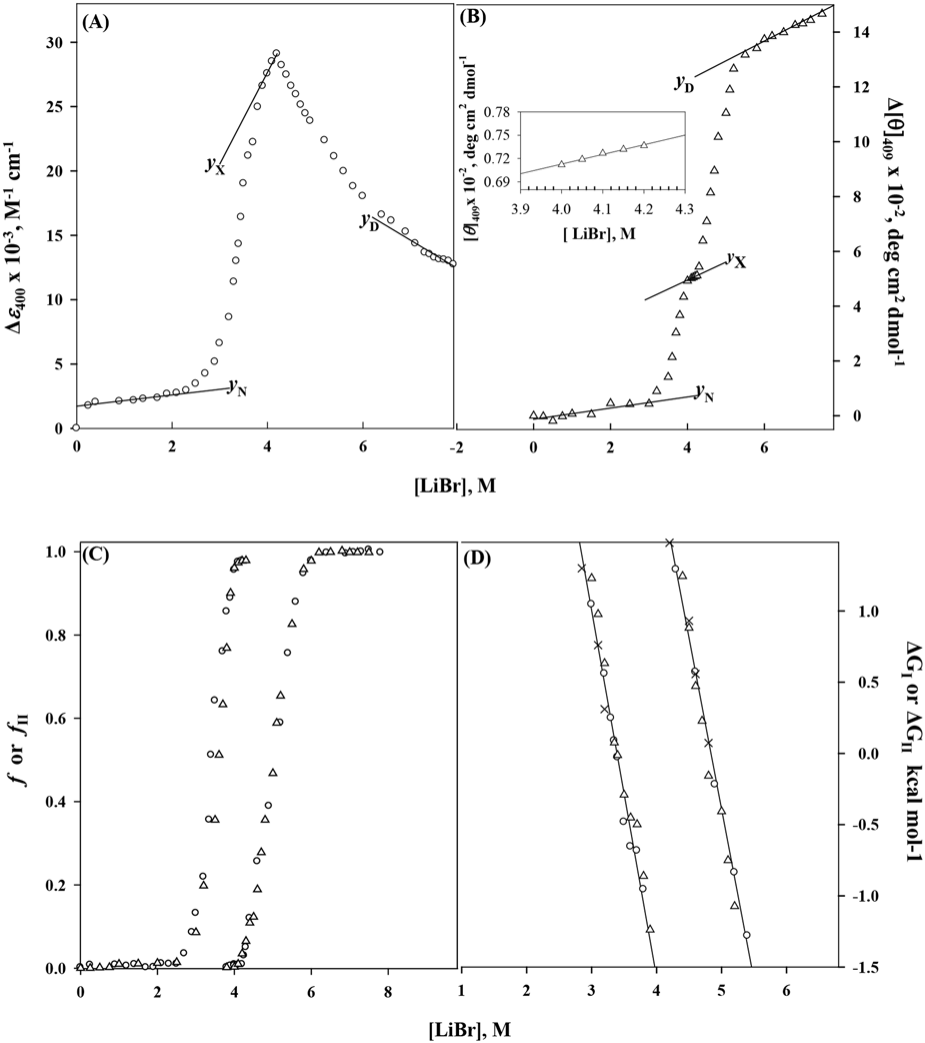
LiBr-induced denaturation of cyt-*c* at pH 6.0 and 25°C. Equilibrium unfolding transition curves are monitored by Δ*ε*_400_ (A) and Δ[*θ*]_409_ (B). Panels (C) and (D) show the normalized transition curves and plots of Δ*G*_I_ and Δ*G*_II_ as function of [LiBr], respectively. The inset in panel (B) shows the plot for the dependence of the optical property of X state (*y*_X_) on [LiBr] on the expanded scale. Filled symbol in panel (D) represents Δ*G*_I_ (or Δ*G*_II_) determined from heat-induced denaturation in the presence of a given [LiBr].

LiBr-induced denaturation curves were monitored by observing changes in the absorption at 400 nm (Δ*ε*_400_) and CD measurement at 409 nm ([*θ*]_409_) (Fig. 1). As evident from Figs. 1A and 1B, denaturation transition monitored by Δ*ε*_400_ and Δ[*θ*]_409_ consists of two distinct processes along the denaturation pathway, N(native) ↔ D(denatured). The first transition (N↔X, where X is the intermediate state) is centered in [LiBr] (molar concentration of LiBr) range from 0 to 4.1 M. The second transition X↔D occurs in [LiBr] range of 4.2–8 M. We can measure the *y*_N_ (optical property of protein molecules in the native state) and *y*_D_ (optical property of protein molecules in the denatured state) which are well defined. Using a linear least-squares analysis, their dependence on [LiBr] was determined by fitting the data in the pre- and post-transition regions, respectively. Solid lines shown in the Fig. 1 depicts the dependencies of *y*_N_ and *y*_D_ on [LiBr]. Fig. 1A shows that the X state exists in a very narrow denaturant concentration range, and the dependence of *y*_X_ (optical property of the X state) on [LiBr] cannot be determined from the isothermal results. For the determination of *y*_X_ dependence on [LiBr], we have used the procedure described earlier for LiCl- and CaCl_2_-induced denaturations [7]. The dependence of *y*_X_ on [LiBr] obtained from the heat-induced denaturation curves of cyt-*c* in the presence of LiBr in the concentration range 2.5–3.8 M, is described by a straight line (Fig. 1A). The inset in Fig. 1B shows the [LiBr] dependence of the CD at 409 nm of protein molecules in X state. Interestingly, it has been observed that both transitions, N ↔ X and X ↔ D, are reversible.

Assuming that the process N↔X, designated here as transition I, follows a two-state mechanism, results shown in Figs. 1A and 1B were used to determine values of *f*_I_ (fraction of protein molecules in the intermediate state) and *ΔG*_I_ (Gibbs energy change associated with transition I) using the relations,

$$f_{I}\;=\;(y-y_{N})\;/\;(y_{X}-y_{N}\;)$$ (4A)

$$\Delta G_{I}\;=\;-RT\;\text{ln}[(y-y_{N})\;/\;(y_{X}-y)]$$ (4B)

where *R* is the universal gas constant, *T* is the temperature in Kelvin, and *y* is the observed optical property corresponding to transition I. Values of *f*_I_ and those of Δ*G*_I_ in the range -1.3 kcal mol^-1^ ≤ Δ*G*_I_ ≤ 1.3 kcal mol^-1^ are plotted as a function of [LiBr] in Figs. 1C and 1D, respectively. The Δ*G*_I_ versus [LiBr] plot was analyzed for Δ*G*_I_^0^ (Δ*G*_I_ value at zero [LiBr] and *m*_I_, the slope (δΔ*G*_I_/ δ[LiBr]), using the relation,

$$\Delta G_{I}=\Delta G_{I}0-m_{I}[LiBr]$$ (5)

Table 1 shows values of Δ*G*_I_^0^, *m*_I_ and *C*_mI_, the midpoint of transition I (= Δ*G*_I_^0^/*m*_I_).

**Table 1:** Thermodynamic parameters characterizing the weak salt denaturants-induced denaturations of cyt-*c* at pH 6.0 and 25 ± 0°C.

Denaturant	Transition	Δ*G*_I_^0^ or Δ*G*_II_^X,^ ^a^	*m*_I_ or *m*_II_	*C*_mI_ or *C*_mII_
		kcal mol^-1^	kcal mol^-1^M^-1^	M
LiBr	N ↔ X	8.8±0.5	-2.2±0.2	3.4±0.1
	X ↔ D	1.4±0.3	-2.6±0.2	4.8±0.1
LiCl	N ↔ X	9.4±0.1	-1.9±0.1	4.9±0.1
	X ↔ D	1.1±0.1	-1.5±0.2	6.9±0.1
LiClO_4_	N ↔ X	9.3±0.2	-6.1±0.1	1.5±0.1
	X ↔ D	1.7±0.1	-2.7±0.2	1.5±0.1

^a^ Δ*G*_II_^X^ is the value of Δ*G*_II_ at [denaturant] where X state exists

Assuming that the process X ↔ D, designated as transition II, is also of a two-state type, results shown in Figs. 1A and 1B were used to determine values of *f*_II_ (fraction of molecules in the D state) and Δ*G*_II_ (Gibbs energy change associated with transition II) using the relations,

$$f_{II}\;=\;(y-y_{X})\;/\;(y_{D}-y_{X})\;$$ (6A)

$$\Delta G_{II}\;=\;-RT\ln[(y-y_{X})\;/\;(y_{D}-y)]$$ (6B)

where *y* is the observed optical property corresponding to transition II and *y*_D_ is the optical property of the denatured protein molecule. Fig. 1C shows the plot of *f*_II_ versus [LiBr]. Values of Δ*G*_II_ in the range -1.3 kcal mol^-1^ ≤ Δ*G*_II_ ≤ 1.3 kcal mol^-1^ are plotted as a function of [LiBr] in Fig. 1D. It is seen in this figure that the plot of Δ*G*_II_ versus [LiBr] is linear. A linear least-squares analysis was used to obtain values of Δ*G*_II_^0^ and *m*_II_ using the relation,

$$\Delta G_{II}=\Delta G_{II}^{0}-m_{II}\left[LiBr\right]$$ (7)

where, subscript II represents the fact that these parameters correspond to transition II, and “0” represents the value at 0 M LiBr. Values of Δ*G*_II_^X^, the value of Δ*G*_II_ at 4.2 M LiBr, *m*_II_ and *C*_mII_ (= Δ*G*_II_^0^/ *m*_II_) are given Table 1.

**Fig 2:**
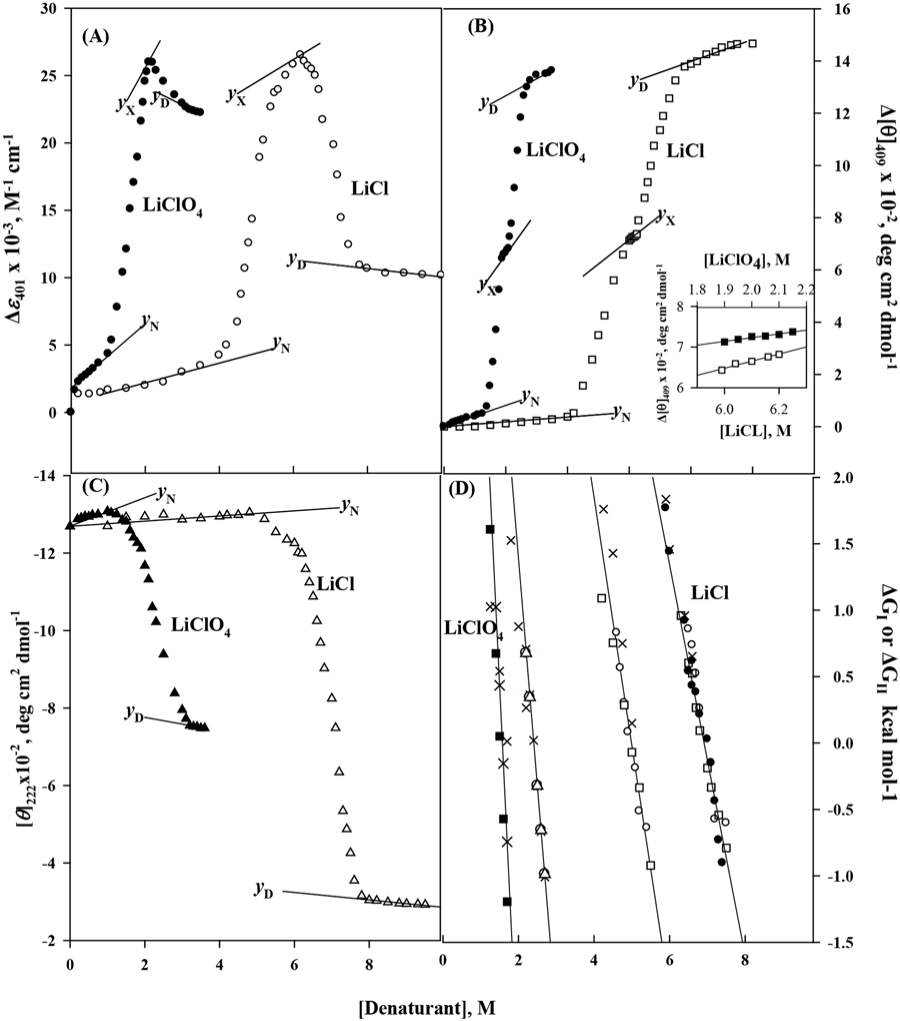
Equilibrium denaturation curves of cyt-*c* in the presence of LiCl (Ο) and LiClO_4_ (Δ) at pH 6.0 and 25°C. Denaturation was monitored by Δ*ε*_400_ (A), Δ[*θ*]_409_ (B) and [*θ*]_222_ (C). Inset in panel (B) shows the plot for the dependence of the optical property of the X state (*y*_X_) on [denaturant] on the expanded scale. Panel (D) shows plots of Δ*G*_I_ and Δ*G*_II_ versus [denaturant]. In this panel filled symbols are obtained from the studies of thermal denaturation in the presence of weak salt denaturants.

LiCl-induced and LiClO_4_-induced denaturations of cyt-*c* were measured by observing changes in Δ*ε*_401_, [*θ*]_409_ and [*θ*]_222_. We have observed that the LiCl- and LiClO_4_-induced denaturations of cyt-*c* are biphasic transitions when monitored by observing changes in Δ*ε*_401_ and [*θ*]_409_ (Figs. 2A and B) whereas, a single step unfolding was observed in case of [*θ*]_222_ measurements (Fig. 2C). Furthermore, denaturations of cyt-*c* induced by LiCl and LiCO_4_, are reversible. Denaturation curves were analyzed for thermodynamic parameters associated with transitions N↔X and N↔D using equations (4–7) in a manner similar to that used for the analysis of LiBr-induced denaturation curves. Values of the thermodynamic parameters for transition I and II are given in Table 1.

**Fig 3:**
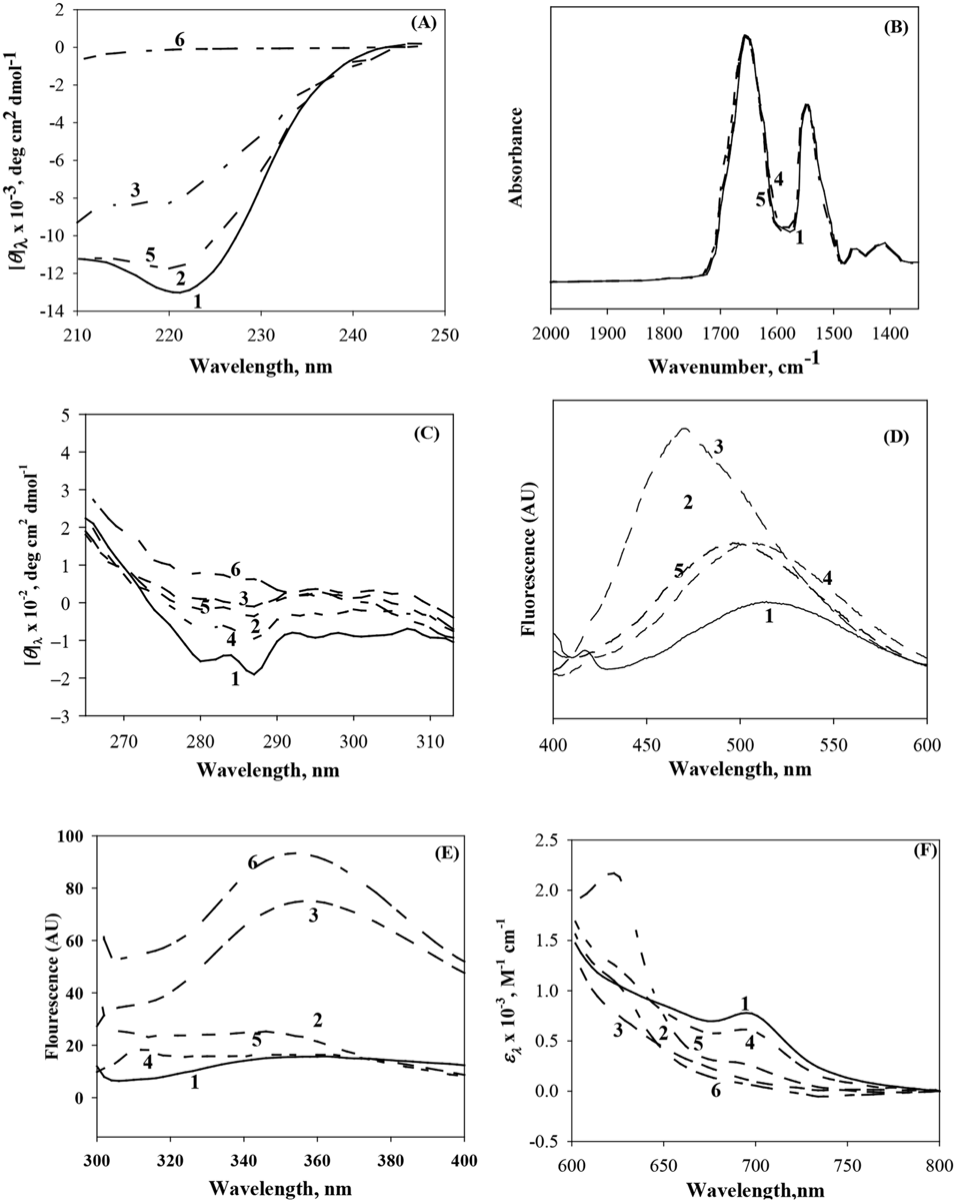
Structural characterization of various states of cyt-*c*. In this figure curve numbers have the same meaning in all panels: 1, native state; 2, LiCl-induced MG state in the presence of 6.2 M concentration; 3, LiClO_4_-induced MG state in the presence of 1.9 M concentration; 4, LiBr-induced MG state in the presence of 4.2 M concentration; 5, 0.5 M NaCl-induced at pH 2.0; and 6, GdmCl-induced denatured state. Panels A and C show the far-UV and near-UV CD spectra. Panel B shows FTIR spectra. Panels D and E show ANS and Trp-fluorescence spectra. Panel F shows near-IR spectra.

The far-UV CD spectrum was measured to observe the change in secondary structure of cyt-*c* in X-state induced by different solvent conditions [39,40]. Fig. 3A shows the far-UV CD measurements of the native state (curve 1), X-state induced by LiCl (curve 2) and LiClO_4_ (curve 3), 0.5 M NaCl-induced MG state at acidic pH (curve 5) and GdnHCl-induced denatured state (curve 6) of cyt-*c*. It is seen in this figure that the far-UV CD spectrum of X state induced by LiCl and 0.5 M NaCl-induced MG state at acidic pH is closer to that of the native state of cyt-*c* indicating the retention of secondary structure in these states. But the X-state induced by LiClO_4_ has lost some of its secondary structure content as shown in Fig. 3A. The cyt-*c* solution containing even a small amount of LiBr shows very large noise signal due to elevation of the HT voltage in the far-UV region. Hence CD in this region cannot be used to determine the secondary structure composition.

FTIR spectroscopy is a sensitive probe to measure the conformational changes of proteins [41], for the major peak between 1700 and 1600 cm^-1^_,_ the so called amide I band, is composed of several underline components arising from secondary structural elements (α-helix, β-sheet and β-turns) and random coil [42,43]. Fig. 3B shows the primary FT-IR spectra of the native, 0.5 M NaCl-induced MG state at acidic pH and LiBr-induced intermediate state (X) of cyt-*c*. It is seen in this figure that the spectra of X state induced by LiBr and MG state induced by NaCl at acidic pH almost overlap with the spectrum of the native state. The spectrum of the native shows the major peak amide I band between 1700 and 1600 cm^-1^ and amide II band between 1600 and 1500 cm^-1^ which are in profound agreement with those reported earlier for other homologue of cyt-*c* [44].

The near-UV CD spectrum (260–320 nm) was used to determine changes in the tertiary structure of cyt-*c*. The negative peak at 287 nm in the near-UV CD spectrum can be ascribed to the tyrosyl side chain [45]. Results of measurements of the near-UV CD spectra of cyt-*c* under different experimental conditions are shown in Fig. 3C. As shown in Table 2, [*θ*]_287_ follows the trend native state > LiBr-induced X-state > LiCl-induced X-state ~ 0.5 M NaCl-induced MG state at acidic pH > LiClO_4_-induced X state.

**Table 2:** Structural and thermodynamic properties of various molten globule (MG) states of cyt-*c* at 25°C and pH 6.0.

	[*θ*]_287_	[*θ*]_222_	*ε*_695_	*R*_h_	*R*_G_	CF	Δ*G*_I_^0^	Δ*G*_II_^X,d^
	(deg cm^2^ dmol^-1^)	(deg cm^2^ dmol^-1^)	(M^-1^ cm^-1^)	(Å)	(Å)	(%)	kcal mol^-1^	kcal mol^-1^
Native	-190	-12,886	778	16.5	12.7	—	—	—
LiBr	-96	—	613	18.2	14.0	90	8.8	1.4
LiCl	-46	-12,204	204	19.1	14.8	85	9.4	1.1
LiClO_4_	-11	-7,767	116	21.2	16.4	75	9.3	1.7
Acid	-36	-11,497	266	19.2	14.8	85	9.1	1.7

The MG state of a protein has more hydrophobic surfaces as compared to its native state as some internal non-polar groups of the protein become exposed to water and hence MG state can strongly bind non-polar molecules from solution than the native state [20]. ANS is environmentally sensitive probe and has been used to detect hydrophobic sites on the surface of proteins [46,47]. The fluorescence of the protein will show increase in its intensity and blue shift in the emission maximum on binding with exposed hydrophobic clusters which are known to be present in the molten globule [47,48]. Fig. 3D shows the ANS fluorescence of X states of cyt-*c* under different solvent conditions. The fluorescence quantum yield of cyt-*c* in LiBr-induced X state shows increase it its intensity in comparison to that of cyt-*c* in the native state though there is marginal blue shift. Cyt-*c* in LiCl-induced X state shows increase in fluorescence quantum yield as well as a blue shift in fluorescence emission maximum from 507 to 490 nm. ANS fluorescence showed an increase in emission maximum (507 nm) and blue shift (460 nm) upon binding to X state of cyt-*c* induced by LiClO_4_ and 0.5 M NaCl-induced MG state at acidic pH.

The change in the tertiary structure of the protein can also be evaluated using tryptophan fluorescence [49]. As shown in Fig. 3E, the fluorescence spectra of the native state, X state induced by LiBr and LiCl, and 0.5 M NaCl-induced MG state at acidic pH are very similar. The LiClO_4_-induced X state and GdmCl-induced D state show increase in fluorescence intensity.

Fig. 3F shows the near-IR absorption spectra of the native state, X states induced by weak salt denaturants and 0.5 M NaCl-induced MG state at acidic pH. The absorbance band at 695 nm is an indicative of the presence of a native Met80 sulfur-iron bond [50,51]. The LiBr-induced X state is in low spin state and has absorption band at 695 nm as that of the native state of cyt-*c*. The extinction coefficient values at 620 nm and 695 nm for the native and acid-induced MG states of cyt-*c* (Table 2) are in excellent agreement with those of other homolog of cyt-*c* reported earlier [12,52]. As shown in Table 2, the spectral intensity at 695 nm follows the trend native state > X-state in LiBr ~ 0.5 M NaCl-induced MG state at acidic pH > X-state in LiCl > X-state in LiClO_4_.

Dynamic light scattering (DLS) experiments were performed to obtain the hydrodynamic radius (*R*_h_) of each state of cyt-*c*. Using equation (3), we have observed a value of 1.5 nm for the theoretical *R*_h_ of cyt-*c*, which is, within experimental errors, identical to that obtained experimentally. The values of *R*_h_ of cyt-*c* were measured under different solvent conditions, which are given in Table 2. It is interesting to note that the *R*_h_ of the native protein and that of the MG states in different solvent conditions are almost similar to those reported earlier [20]. The compactness factor of the MG state and X state in a given solvent condition was determined using equation (2) with *R*_h_ values of the native state, MG (X) states and denatured states under different solvent conditions. Table 2 shows *R*_g_ values of X state under different solvent conditions which are calculated using the relation, *R*_g_ = *ρ R*_h_, where *ρ* = (3/5)^1/2^ for a solid sphere [53].

### Effect of Temperature on N ↔ X Equilibrium

**Fig 4:**
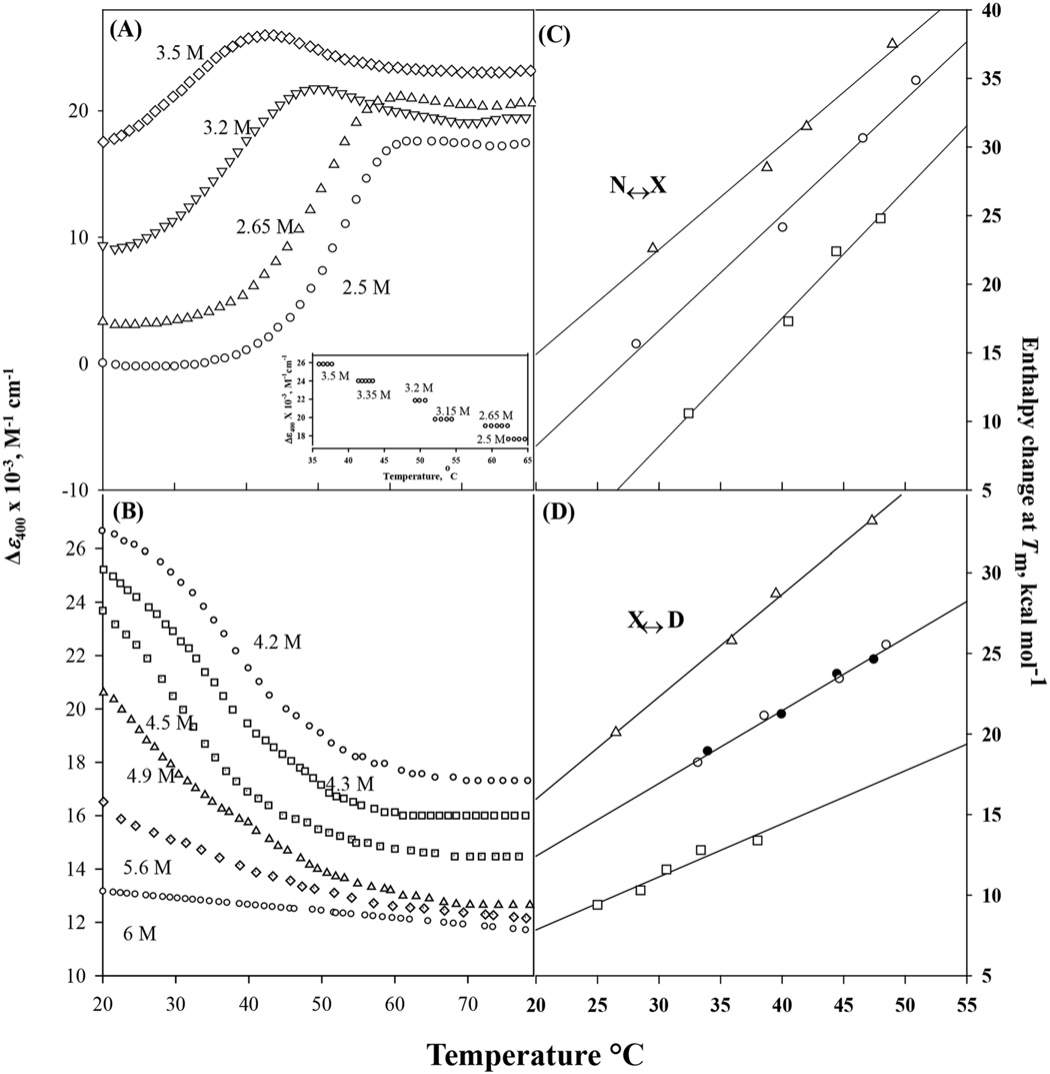
Thermal denaturation of cyt-*c* in the presence of different LiBr concentrations measured by Δ*ε*_400_ at pH 6.0. Panels A and C represent N↔X transition, and panels B and D represent X↔D transition. All curves are not shown to maintain clarity. Inset in panel (A) shows plots of *y*_x_, the optical property of X state on temperature at different LiBr concentrations. Plots of Δ*H*_m_ versus *T*_m_ of cyt-*c* in the presence of LiCl (Ο), LiClO_4_ (□) and LiBr (Δ) obtained from Δ*ε*_400_ measurements for N↔X process (C), while panel (D) shows (Δ*H*_m_, *T*_m_) data for the process X↔D. Filled symbol represents data point obtained from [*θ*]_222_ measurements.

We have studied the effect of temperature on cyt-*c* in the presence of LiBr in the concentration range 2.5–3.5 M in which N↔X equilibrium exists at 25°C (see Fig. 1A). It has been observed that the thermal denaturation of the protein is reversible at all LiBr concentrations. Fig. 4A shows typical heat-induced transition curves in this [LiBr] range. It is clearly observed in this figure that cyt-*c* in the presence of a fixed [LiBr] undergoes a biphasic transition. However, the transition occurring at higher temperature range is not completed even at 85°C.

Assuming that the first transition curve of cyt-*c* at a given [LiBr] shown in Fig. 4A, represents a two-state process, N↔X (transition I), one can determine Δ*G*_I_ as a function of temperature using equation (4B), provided temperature dependencies of *y*_N_ and *y*_X_ are known at each [LiBr]. Inset of Fig. 4A shows plots Δ*ε*_400_ of the protein in X state as a function of temperature at different LiBr concentrations. It is seen in this figure that *y*_X_ can be observed in a very narrow temperature range, and at a given [LiBr] it does not depend on temperature. However *y*_X_ shows dependence on [LiBr]. The relation describing this [LiBr]-dependence of *y*_X_ was determined, and it was used to draw the solid line showing *y*_X_ dependence on [LiBr] in Fig. 1A.

To determine the dependency of *y*_N_ of cyt-*c* on the composition variables ([LiBr] and temperature), the native protein was heated in the presence of 0.1, 0.4, 0.9 and 1.5 M of LiBr, and Δ*ε*_400_ was measured as a function of temperature. Our observations suggest that *y*_N_ depends on both temperature and [LiBr]. Using these observations on *y*_N_, *y*_X_, and values of *y* (Δ*ε*_400_) of the heat-induced transition between N and X states (Fig. 4A), Δ*G*_I_ was determined at each temperature with the help of equation (4B). At each [LiBr] value of Δ*G*_I_ in the range-1.3 kcal mol^-1^ ≤ Δ*G*_*I*_ ≤ 1.3 kcal mol^-1^ in the transition region were plotted against temperature, and these plots (stability curves not shown) were analyzed to determine the value of *T*_m,I_, the midpoint of transition I and Δ*H*_m,I_, the enthalpy change at *T*_m,I_ using the procedure described earlier [54]. Values of *T*_m,I_ and Δ*H*_m,I_ at different concentrations of LiBr are shown in Fig. 3. A linear least-square analysis of Δ*H*_m,I_ versus *T*_m,I_ plot (Fig. 4C) gave the value of 0.76 ± 0.04 kcal mol^-1^ K^-1^ for Δ*C*_p,I_, the constant-pressure heat capacity change (= (∂Δ*H*_m,I_/ ∂*T*_m,I_)_p_) associated with N ↔ X process (transition I) induced by LiBr. Values of *T*_m,I_, Δ*H*_m,I_, and Δ*C*_p,_ were used for determining Δ*G*_I_ at a given [LiBr] and 25°C using the relation,

$$\Delta G_{I}=\Delta H_{m,I}[(T_{m,I}-298.15)/{\mathrm{Tm}}_{,I}]-\Delta C_{p,I}[(T_{m,I}-298.15)+298.15\text{ln}(298.15/T_{m,I})]$$ (8)

### Effect of Temperature on X↔ D Equilibrium

We have measured the heat-induced denaturation curves (plot of Δ*ε*_400_ versus temperature) of cyt-*c* in the presence of LiBr, in the concentration range 4.2–6.0 M in which the protein exhibits X↔D equilibrium at 25°C (see Fig. 1A). We have observed that the thermal denaturation of the protein is reversible at all LiBr concentrations (Fig. 4B). Moreover, the optical property of the denatured protein, *y*_D_, shows a dependence on temperature and [LiBr]. The relation describing this temperature dependence of *y*_D_ at different denaturant concentrations was determined.

Assuming that heat-induced denaturation (transition II) of cyt-*c* in the presence of LiBr follows a two-state mechanism, the transition curve presented in Fig. 4B was analyzed for Δ*G*_II_, at each temperature and [LiBr]. We have constructed stability curves (Δ*G*_II_ versus *T*) using equation (6B) with known dependence of *y*_X_ and *y*_D_ on [LiBr] and *T*. The stability curves (plots not shown) were analyzed for Δ*H*_m,II_ and *T*_m,II_ values. Values of these thermodynamic parameters at different concentrations of LiBr are shown in Table 3. A linear least-squares analysis of Δ*H*_m II_ versus *T*_m,II_ plot (Fig. 4D) gave the value of 0.63 ± 0.01 kcal mol^-1^ K^-1^ for Δ*C*_p,II_, the constant-pressure heat capacity change (= (∂Δ*H*_m,II_/ ∂*T*_m,II_)p) associated with X↔D process (transition II) induced by LiBr. At a given [LiBr], values of *T*_m,II_, Δ*H*_m,II_, and Δ*C*_p,II_ were used for determining Δ*G*_II_ at that given [LiBr] and 25°C using the relation,

**Table 3:** Comparison of thermodynamic parameters of cyt-*c* in the presence of different denaturants at pH 6.0 and 25^o^C^a^,.

Denaturant	Transition	Δ*G*_D_^0^ or Δ*G*_I_^0^ Δ*G*_II_^X^	Δ*H*_m_^0^ or Δ*H*_m, I_^0^ or Δ*H*_m, II_^X^	Δ*C*_p_ or Δ*C*_p, I_ or Δ*C*_p, II_
		kcal mol^-1^	kcal mol^-1^M^-1^	kcal mol^-1^K^-1^
GdmCl^b^	X ↔ D	9.2±0.6	98±3	1.30±0.1
Urea^c^	X ↔ D	9.6±0.4	95±4	1.20±0.1
DSC^c^	X ↔ D	9.1±0.3	91±4	1.23±0.1
LiBr	N ↔ X	8.8±0.5	94±4	0.76±0.04
	X ↔ D	1.5 ±0.3	27±3	0.63±0.01
LiCl	N ↔ X	9.5±0.2	96±4	0.84±0.05
	X ↔ D	1.5±0.1	23±2	0.45±0.03
LiClO_4_	N ↔ X	9.6±0.2	82±4	0.93±0.06
	X ↔ D	1.3±0.1	17±3	0.32±0.04

^a^ A‘±’ with each parameter represents an error from the mean of errors from the triplicate measurements.

^b^ Obtained from [31].

^c^ Obtained from [63].

Δ*G*_D_^0^, Δ*H*_m_^0^ and Δ*C*_p_ represent N ↔ D process in the absence of the chemical denaturant. Δ*G*_I_^0^, Δ*H*_I_^0^ and Δ*C*_p,I_ represent N ↔ X process in the absence of the chemical denaturant. Δ*G* ^X^_II_, Δ*H* ^X^_II_ and Δ*C*_p,II_ represent X ↔ D process in the presence of X M weak salt denaturant.

$$\Delta G_{II}=\Delta H_{m,II}[(T_{m,II}-298.15)/T_{m,II}]-\Delta C_{p,II}[(T_{m,II}-298.15)+298.15\ln(298.15/T_{m,II})]$$ (9)

### Effect of Temperature on LiCl- and LiClO_4_-induced denaturation of cyt-*c*

We have also studied the effect of temperature on N↔X and X↔D equilibria induced by LiCl and LiClO_4_ by measuring changes in Δ*ε*_400_. It was observed that the thermal unfolding of cyt-*c* in the presence of different concentrations of LiCl and LiClO_4_ undergoes a biphasic transition. The thermal unfolding transition curves (not shown) was analyzed using the method as the one used for analyzing thermal transition curves of LiBr shown in Fig. 4. Results of this analysis are shown in Fig. 4C, D and Table 3.

## Discussion

A protein synthesized at ribosome folds into the unique native state to attain its physiological function in a hierarchical manner. During protein folding there is progressive stabilization of structures in steps which leads to accumulation of intermediate states of different conformations [55]. Previously it was thought that small globular proteins exhibit two-state equilibrium unfolding transitions due to lack of stable intermediate state(s) [56]. The unstable intermediate can be stabilized sufficiently relative to the native state by mutation [57,58] or by a change in solvent conditions to accumulate in the course of folding transitions [7,13,14,30,59]. Thus, two-state transitions of protein folding can be converted into three (or more)-state pathways. Compact intermediates of proteins have been observed under both transient and equilibrium conditions.

One such intermediate called molten globule state which is found on the unfolding pathways of many globular proteins, has common structural characteristics: (i) the presence of a pronounced amount of secondary structure, (ii) the absence of most of the specific tertiary structure produced by tight packing of side chains, (iii) compactness of a protein molecule with a radius of gyration 10–30% higher than that of the native state, and (iv) presence of a loosely packed hydrophobic surface accessible to solvent [20,60]. We have previously reported that MG state(s) of cyts-*c* from different sources can be induced upon denaturation by weak salt denaturants (LiCl, CaCl_2_ and LiClO_4_) at physiological pH. Interestingly, there is an apparent uniqueness in the structural integrity of the MG states under different solvent conditions. Therefore, we have designed a strategy to evaluate the extent of structural fluctuation of equilibrium MG states under physiological condition. To achieve this goal, we have investigated properties of MG states of cyt-*c* obtained in various solvent conditions (different weak salt denaturants that differ in size and in exclusiveness based on Hoffmeister series). We observe that denaturation of cyt-*c* by weak salt denaturants, LiBr, LiCl and LiClO_4_ (25°C and pH 6.0), follows a biphasic (N↔X↔D) process due to the accumulation of X states (Figs. 1A-B and 2A-B). X states were found to accumulate predominantly at 4.2 M, 6.2 M and 1.9 M concentrations of LiBr, LiCl and LiClO_4_, respectively.

It can be seen in the Fig. 3A that the far-UV CD spectra of LiCl-induced X state and 0.5 M NaCl-induced MG state (pH 2.0) are almost overlapping with the native state spectrum suggesting that X state induced by LiCl has a structural characteristic of molten globule, i.e., the native secondary structure is retained in X state. But in case of LiClO_4_-induced X state there is less secondary structure in comparison to that of the native state. We could not measure secondary structural content of the protein in the presence of LiBr from the far-UV CD due to technical difficulties. However, we measured the IR-spectra for the native, LiBr-induced X state and 0.5 M NaCl-induced MG of cyt-*c*, which are almost identical (Fig. 3B). Hence the intermediate state X induced by LiBr has one of the structural characteristics of MG state, namely, the presence of most of the secondary structure that the native protein originally had [60].

Due to side chain fluctuation in the MG state, its near-UV spectra are dramatically reduced in comparison to the native one, which suggests a virtual absence of a rigid tertiary structure [12,20,60]. It can be seen in Fig. 3C that the CD in the near-UV region of LiBr-induced-X state shows more negative CD at 282 and 289 nm in comparison to LiCl-, LiClO_4_- and 0.5 M NaCl-induced X states. These findings reveal that the exposure of aromatic side chain is less in LiBr-induced X state in comparison to LiCl-, LiClO_4_- X states and 0.5 M NaCl-induced MG state. Hence, X states induced by various solvent conditions have lost its native tertiary structure. However, this loss is less than that occurring in the presence of concentrated GdmCl.

X state of cyt-*c* induced by LiBr shows increase in ANS fluorescence intensity but there is slight blue shift in the emission maximum in comparison to that of the native protein as shown in Fig. 3D. However, X state of cyt-*c* induced by LiCl, LiClO_4_ and 0.5 M NaCl (pH 2.0) show increase in fluorescence intensity as well as a blue shift in emission maximum which follows the trend LiClO_4_ > LiCl > 0.5 M NaCl > LiBr. These observations suggest that the X state induced under different solvent conditions has a MG characteristic, namely, a loosely packed hydrophobic surface exposed to the solvent. It also reveals there is more exposure of hydrophobic surfaces in X state induced by LiClO_4_ and least exposure in the X state induced by LiBr.

We have also observed that the tryptophan fluorescence intensity of LiCl- and LiBr-induced X states is quenched with respect to that of the native protein while the LiClO_4_-induced X state shows an increase in the intensity (Fig. 3E), indicating that there is less exposure of non-polar groups of cyt-*c* to the solvent in the LiCl- and LiBr-induced X state in comparison to LiClO_4_-induced X state. The results of Trp fluorescence indicate that Trp59 in LiBr-induced X state of cyt-*c* is more close to heme in an apolar environment (due to resonance energy transfer to the adjacent heme group).

The absorption band at 620 nm is absent in LiCl-, LiCLO_4_- and LiBr-induced X states but it is present in NaCl-induced MG state (Fig. 3F). The weak charge-transfer band at 695 nm is absent in LiCl-, LiClO_4_- and NaCl-induced X states but it is present in LiBr-induced X state. Though the LiCl- and LiClO_4_-induced X states have low spin state of the native state of cyt-*c*, they cannot retain the Met80-Fe (III) bond of the native state. The presence of Met80-Fe (III) axial bond in LiBr-induced X state reveals that the retention of a direct interaction between the heme and the 70–85 residues polypeptide segment.

One of the most important properties of the MG state is that it is almost compact as the native state. We have estimated the *R*_g_ values of the native and X states of cyt-*c* induced by LiCl, LiClO_4_ and LiBr as shown in the Table 2. It was observed that the X state has *R*_g_ 10–30% larger than the native state, which is one of the common characteristics of MG state. The *R*_g_ values and compactness factor, CF (%) of the X state of cyt-*c* under different solvent conditions are given in Table 2. These observations indicate that the CF (%) of X states follows the trend, LiBr > 0.5 M NaCl-induced MG (pH 2) ~LiCl > LiClO_4_. This comparison led us to believe that the LiBr-induced X state is the most compact while, LiClO_4_—induced X state is the least compact.

On the basis of the above experimental results, the X state of cyt-*c* induced by LiCl, LiClO_4_ and LiBr is MG state as they possess all common structural characteristics of the MG state [20,60]. It can be concluded that the LiBr-induced MG state of cyt-*c* retaining the Met80-Fe(III) axial bond, Trp59-propionate and hydrophobic interactions of the native state is more close to the native state in comparison to the MG states induced by LiCl, LiClO_4_ and 0.5 M NaCl at pH 2.0. The LiCl-induced MG state of cyt-*c* has all the characteristics of the classical MG state of cyt-*c* induced by 0.5 M NaCl under low pH conditions [7,12,61]. But the LiClO_4_-induced MG state of cyt-c shows complete loss in tertiary structure, disturbed secondary structure, more exposure of hydrophobic patches with losing Met80-Fe(III) axial bond and Trp59-propionate interactions of the native state of cyt-*c*. Our findings suggest the existence of heterogeneity of equilibrium intermediates along the unfolding pathway of cyt-*c* as highly ordered (X1), classical (X2) and disordered (X3), i.e. D ↔ X3 ↔ X2 ↔ X1 ↔ N under physiological conditions.

The molten globule state of the protein cyt-*c* is not only structurally quite different from the unfolded and native states, but also represents a new thermodynamic state in addition to the previously known native and denatured states [60]. To analyze N↔X↔D transition for thermodynamic parameters of cyt-*c* at 25°C, two assumptions, namely, two-state mechanisms of weak salt-induced denaturation and a linear dependence of Δ*G*_D_ on [Denaturant] were introduced. One of the criteria to test the validity of a two-state transition is to see whether one gets comparable values of thermodynamic parameters associated with the transition curve monitored by different structural probes. Since values of *f* and Δ*G* obtained from transition curves of different optical properties fall on the same *f* versus [LiBr] (Fig. 1C) and Δ*G* versus [LiBr] (Fig. 1D) plots, respectively, a two-state assumption seems to be valid. A more authentic test for the validity of the two-state assumption is to compare the total Gibbs free energy change associated with transitions I and II (i.e., Δ*G*_I_^0^ + Δ*G*_II_^X^) observed here with that from the differential scanning calorimetric (DSC) measurements for a two-state N↔D transition. It is noteworthy that the value of calorimetric Gibbs free energy change (Δ*G*_D_^0^) for the N ↔ D transition, which is 10 ± 1 kcal mol^-1^ [62,63] is in agreement with that (Δ*G*_I_^0^ + (Δ*G*_II_^X^) obtained from optical methods (Table 1). The correlation between the thermodynamic parameters obtained from DSC and optical measurements reveals that our analysis of denaturation curves is accurate, thermodynamic parameters derived from this analysis are authentic, and each process (transition I and transition II) follows a two-state mechanism. The assumption that Δ*G*_D_ vary linearly with [LiBr] is also justified as values of Δ*G*_I_ (or Δ*G*_II_) estimated from the heat-induced denaturation of cyt-*c* in the presence of LiBr falls on the same line drawn from the denaturation results obtained at 25°C (see • symbols in Fig. 1D) as shown earlier [7]. The results of LiCl and LiClO_4_, shown in Fig. 2D and Table 3, can also be explained on the same line of arguments given for LiBr-induced denaturation.

LiBr-induced denaturation measurements at 25°C (Fig. 1) and heat-induced denaturation measurements in the presence of appropriate concentrations of LiBr (Fig. 4) revealed that the unfolding transition of the MG state of cyt-*c* is highly cooperative and reversible. As shown in Fig. 4D, the plot of Δ*H*_m,II_ and *T*_m,II_ obtained from the heat-induced denaturation of cyt-*c* in the presence of different LiBr concentrations gave the value of Δ*C*_p,II_ as 0.63 ± 0.01 kcal mol^-1^ K^-1^. Extrapolation of Δ*H*_m,II_ and *T*_m,II_ versus [denaturant] plots (not shown) to the concentrations of LiBr in which the protein exists in MG state gave, respectively values of 27.3 ± 3.1 kcal mol^-1^ and 48.8 ± 0.5°C. These values are in excellent agreement with those obtained from DSC measurements; Δ*H*_m,II ~_ 35 kcal mol^-1^ and *T*_m,II_ ~ 50°C [64–66]. Δ*G*_X,II_ value (1.2 kcal mol^-1^) obtained using values of Δ*H*_m,II_, Δ*C*_p,II_ and *T*_m,II_ in equation (9), is in excellent agreement with that from isothermal measurements (see Table 1) as well as from calorimetric measurements (1.33 kcal mol^-1^ at 25°C) [64]. The heat-induced MG ↔ D process of cyt-*c* is a two-state process, for, within experimental errors, identical values of thermodynamic parameters are obtained from two different optical properties of the protein.

Analysis of the reversible heat-induced denaturation transition N ↔ MG in the presence of different LiBr concentrations, gave values of *ΔH*_m,I_ and *T*_m,I_. The plot of Δ*H*_m,I_ and *T*_m,I_ gave the value of Δ*C*_p,I_ = 0.76 ± 0.04 kcal mol^-1^K^-1^ which is in excellent agreement with that obtained from DSC measurements [64,65,67]. Values of Δ*G*_I_ are obtained using the values of Δ*H*_m,I_, *T*_m,I_ and Δ*C*_p,I_ in equation (8), and these Δ*G*_I_ values were plotted against [LiBr]. Extrapolation of the plot to 0 M LiBr gave the value of 8.8 ± 0.5 kcal mol^-1^ for Δ*G*_I_.

A comparison of thermodynamic parameters (Δ*C*_p,I_ and Δ*C*_p,II_) obtained from the heat-induced denaturation of cyt-*c* under different solvent conditions (see Table 3) reveals that Δ*C*_p,I_ follows the trend as LiClO_4_ > LiCl > LiBr, while the Δ*C*_p,II_ follows the trend as LiBr > LiCl > LiClO_4_. These observations suggest that the hydrophobic residues in LiBr-induced MG state are more shielded from water in comparison to the MG state obtained from other solvent conditions. Taking water accessibility of hydrophobic surfaces as a measure of unfolding, the above trend shows that LiClO_4_-induced MG state is more unfolded than those obtained from LiBr, LiCl and 0.5 M NaCl (pH 2.0), which is in excellent agreement with conformational studies under isothermal conditions.

Moench et al. [68] reported that there exist many positively charged amino acid side chains on the surface of cyt-*c* near neutral pH, which function as anion binding sites. The anions of weak salt denaturants (Cl^-^, ClO_4_^-^ and Br^-^) interact with positive charges on the surface of protein (oxidized cyt-*c* bears greater positive charge at neutral pH). According to Sola Group, the chloride ion binds to cyt-*c* to a greater extent in comparison to other anions [69]. Though the perchlorate anions shows the same binding stoichiometry with chloride ion [70–72], ClO_4_^-^ which can acts as a chaotrope, has more preferential interaction with cyt-*c* leading to its more unfolding of cyt-*c* [14].

There is a decrease in Δ*S*_m,II_ associated with MG ↔ D (not shown here). As the conformational entropy of MG state cannot be higher than that of D state, the observed entropy suppression must arise from the entropy of water. The origin of the change in water entropy is hydrophobic effect [73] so that this effect is one interaction stabilizing MG state. It seems that hydrophobic effect seemingly plays a role for the more compact structure of LiBr-induced MG state in comparison to LiCl-induced MG state. Moreover, the MG state retains the secondary structure present in the native state (see Fig. 3A and 3B). These results indicate that the MG state of cyt-*c* is stabilized by hydrophobic interactions and secondary structure.

It has been reported that unfolding of the 70–85 loop foldon in the low energy unfolding intermediate identified in the native-state by HX studies (which includes unfolding of the 40–75 loop foldon as well), involves loss of Met80-heme coordination and increased disorder in the nearby loop structure of cyt-*c* [74,75]. Santucci et al. [13] reported that the hydrophobic core contributes mostly to the stability of MG state, while the contribution of the highly flexible loop region is responsible for the different spectroscopic and redox properties. It is evident from the Table 2 that the Gibbs energy change is, within experimental errors, the same for the transition between the N state and different MG states. This means that different MG states of cyt-*c* retain the hydrophobic core. The Met80-Fe(III) axial bond and Trp59-propionate interaction are responsible mainly for the different absorption spectroscopic properties of different MG states of the protein. The Met80-Fe(III) axial bond and Trp59-propionate interaction of the native state are present in LiBr-induced MG state hence this MG state is closer to the native state than the other MG states of cyt-*c*. The LiBr-induced MG state is very similar to the low energy foldon state, posited from the HX evidence and the equilibrium M state detected by heme band CD and MCD spectroscopy [74,76,77].

It has been reported earlier that the pH-induced transition of cyt-*c* between pH 7 and 13 involves four folding intermediates between well characterized folded native state III and unfolded state D i.e. III ↔ III* ↔ (IV_a_ ↔ IV_b_) V ↔D [19,78]. The MG state induced by LiBr at high ionic strength bears similarity with state III* (induced at low ionic strength) as it shows Met80-Fe axial bond and secondary structure intact, and change in the tertiary structure. The MG state induced by LiCl and LiClO_4_ may represent the alkaline transition intermediate states IV and V as these do not retain the Met80-Fe axial bond and tertiary structure though the secondary structure is intact. Our results confirm earlier report that both the increase of the ionic strength and anion binding to the protein surface can have a significant influence on the Met80-Fe axial bond, which is a major determinant of the redox of cyt-*c* [79].

## Conclusions

The MG state could be divided into three major classes: highly ordered, classical and disordered [80]. Taken the specific tertiary structure as the orderly mark, the classical MGs that exhibit a reduced tertiary structure and increased fluctuations of side chain are between highly ordered and disordered categories [81]. From our thermodynamic and conformational studies of weak salt denaturant-induced MG states, we conclude that LiCl-induced and anion-induced (pH 2.0) MG states which have native-like secondary structure, compactness and reduced tertiary structure, qualify for the classical MG state. On the other hand, LiClO_4_-induced MG state belongs to the disordered MG state as it has both disturbed secondary and tertiary interactions. Moreover, the LiBr-induced MG state can be categorized as highly ordered as it has native-like secondary structure, compactness and nearly native like tertiary structure.

## Supporting Information

S1 Fig LiBr-induced denaturation of cyt-*c*. Representative difference absorption spectra (A) and difference CD spectra (B) in the Soret region (370–500 nm) and difference absorption spectra (C) in the near-IR region (660–750 nm) in the presence of different concentrations of LiBr.
(TIF)

S2 Fig LiCl-induced denaturation of cyt-*c*. Representative difference absorption spectra (A) and difference CD spectra (B) in the Soret region (370–500 nm) and difference absorption spectra (C) in the near-IR region (660–750 nm) in the presence of different concentrations of LiCl.
(TIF)

S3 Fig LiClO_4_-induced denaturation of cyt-*c*. Representative difference absorption spectra (A) and difference CD spectra (B) in the Soret region (370–500 nm) and difference absorption spectra (C) in the near-IR region (660–750 nm) in the presence of different concentrations of LiClO_4_.
(TIF)
