# Supplementary figures and images for: Heterogeneity of Equilibrium Molten Globule State of Cytochrome c Induced by Weak Salt Denaturants under Physiological Condition
Source: PLoS One. 2015 Apr 7;10(4):e0120465. doi: 10.1371/journal.pone.0120465 (PMC4388492; doi:10.1371/journal.pone.0120465)

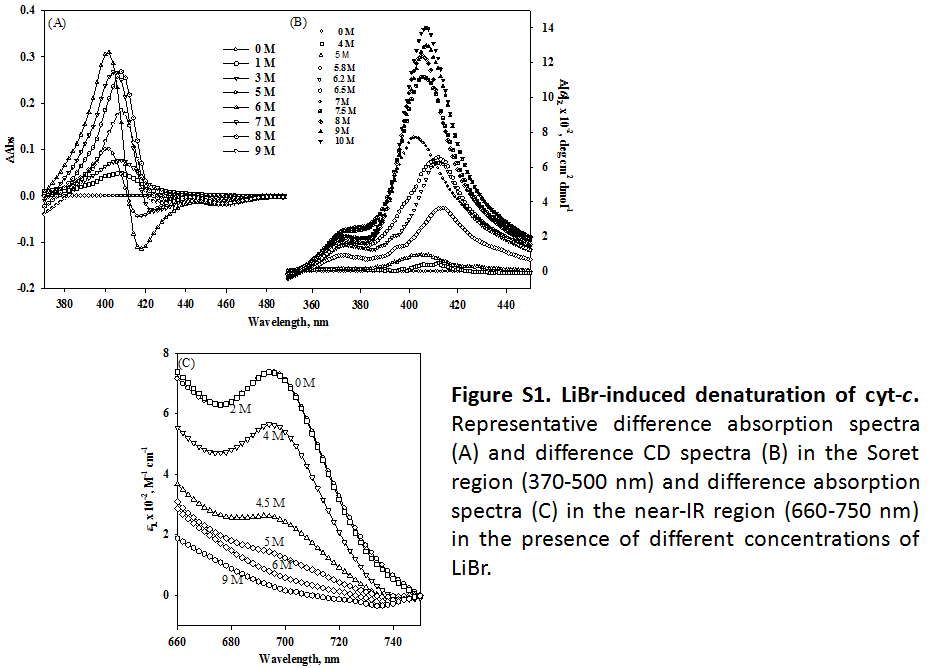

Supplement: S1 Fig — Representative difference absorption spectra (A) and difference CD spectra (B) in the Soret region (370–500 nm) and difference absorption spectra (C) in the near-IR region (660–750 nm) in the presence of different concentrations of LiBr. (TIF) [file pone.0120465.s001.tif]

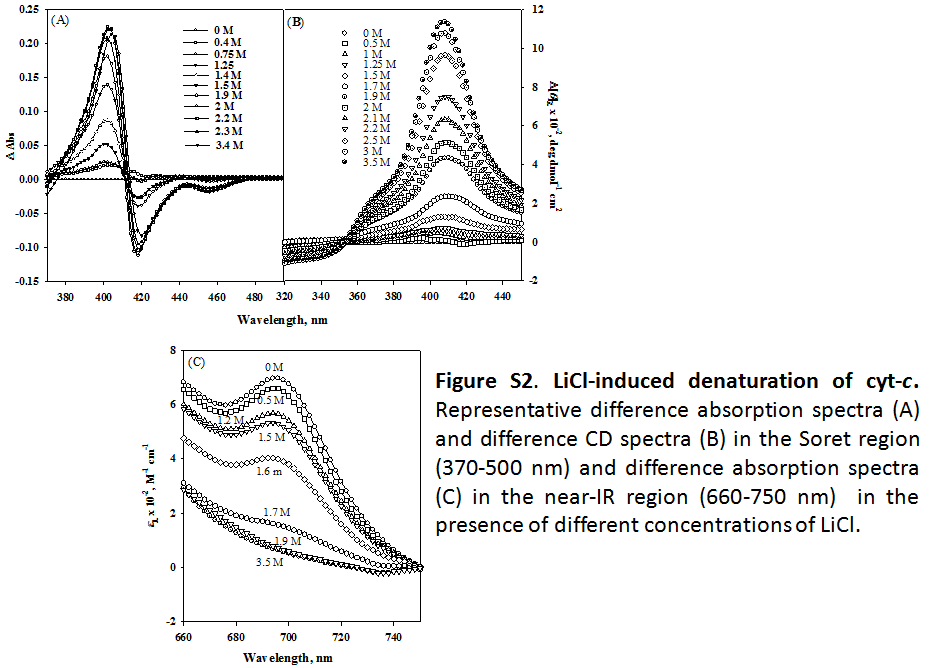

Supplement: S2 Fig — Representative difference absorption spectra (A) and difference CD spectra (B) in the Soret region (370–500 nm) and difference absorption spectra (C) in the near-IR region (660–750 nm) in the presence of different concentrations of LiCl. (TIF) [file pone.0120465.s002.tif]

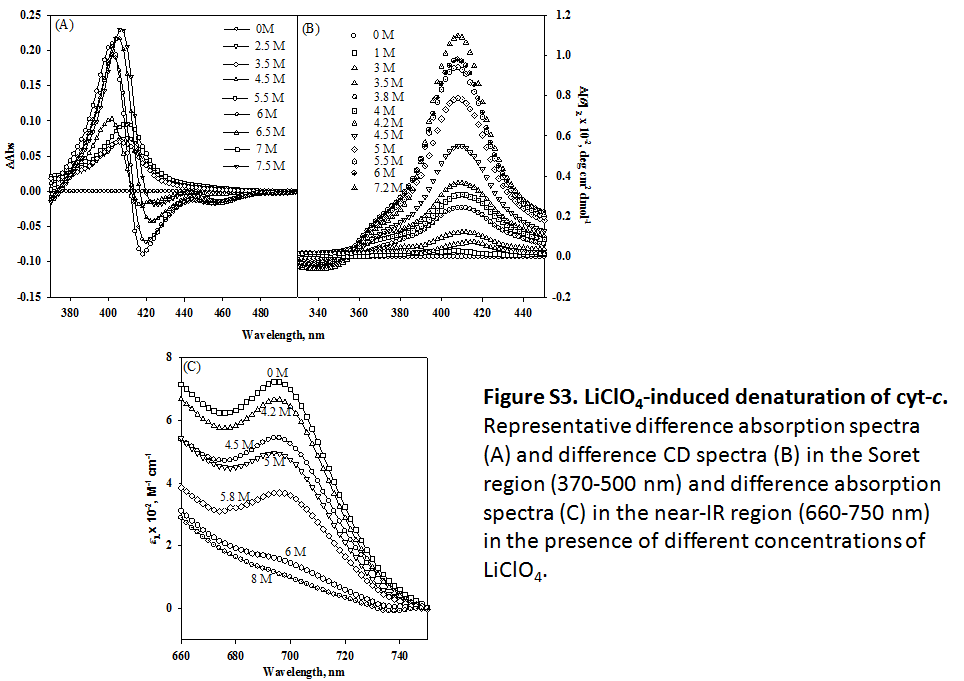

Supplement: S3 Fig — Representative difference absorption spectra (A) and difference CD spectra (B) in the Soret region (370–500 nm) and difference absorption spectra (C) in the near-IR region (660–750 nm) in the presence of different concentrations of LiClO4. (TIF) [file pone.0120465.s003.tif]
